# Normal Levels of Ionized Calcium Despite Persistent Increase in Total Calcium in a Patient With IgA Paraproteinemia

**DOI:** 10.1210/jcemcr/luad163

**Published:** 2023-12-22

**Authors:** Ignacio Portales-Castillo, Abdullah Jalal, Peggy L Kendall, Deborah Parks

**Affiliations:** Department of Medicine, Division of Nephrology, Washington University in St.Louis, St. Louis, MO 63110, USA; Department of Medicine, Division of Nephrology, Washington University in St.Louis, St. Louis, MO 63110, USA; Department of Medicine, Division of Allergy and Immunology, Washington University in St.Louis, St. Louis, MO 63110, USA; Department of Medicine, Division of Rheumatology, Washington University in St.Louis, St. Louis, MO 63110, USA

**Keywords:** hypercalcemia, ionized calcium, IgA, albumin, pseudohypercalcemia

## Abstract

Approximately half of the calcium in the blood circulates in the ionized, free form; which is critical for cellular function. As a result, its levels are tightly regulated by homeostatic mechanisms dependent on hormones such as PTH, vitamin D, and fibroblast growth factor-23. The other half of the total calcium is in a complex with anions, predominantly albumin. Clinically, the levels of albumin are known to influence the relationship of total calcium to free calcium. However, the relevance of changes in other serum proteins on calcium homeostasis is less appreciated. We present the case of a 70-year-old woman who was followed over 5 years with persistently elevated total calcium levels but with normal ionized calcium levels. Her evaluation was notable for IgA paraprotein, which paralleled her history of elevated total serum calcium. Extensive clinical investigations did not reveal hyperparathyroidism or cancer-mediated hypercalcemia. Additional in vitro analyses comparing the plasma containing the IgA paraprotein against a healthy control revealed that a high-molecular-weight IgA paraprotein in the patient has increased capacity to reduce the amount of free calcium in solution, thus providing a direct mechanistic explanation for the clinical findings.

## Introduction

The levels of ionized calcium (Ca^++^) in the blood are tightly regulated by the coordinated actions of hormones including PTH, vitamin D, and fibroblast growth factor-23 and their target organs, which include the parathyroid gland, kidney, bones, and intestines [1]. When the levels of Ca^++^ are low, the calcium-sensing receptor releases tonic suppression on the parathyroid cells to allow for increased secretion of PTH, which in turn releases calcium from the bones and acts on the kidney to form activated vitamin D via an increase in 1alpha-hydroxylase. On the other hand, high levels of Ca^++^ suppress the release of PTH, via activation of the calcium-sensing receptor [1].

Approximately half of the calcium in the blood circulates bound to negatively charged proteins, predominantly albumin, and proportionally less to other proteins including immunoglobulins [2]. The remaining free calcium is critical for cellular function and thus homeostatic mechanisms operate based on levels of Ca^++^ [1].

Changes in serum albumin concentration, serum pH, and total protein affect the proportion of ionized calcium relative to total calcium [3]. Indeed, low levels of total serum calcium with normal ionized calcium are frequently encountered clinically, particularly in patients who are acutely ill, have acid–base disorders, and those with hypoalbuminemia [2]. However, discrepancies in the relative proportion of bound and free calcium caused by other factors beyond albumin and serum pH are harder to study in the clinical setting [4].

Here, we present the case of a 70-year-old woman who was followed over 5 years with new persistently elevated total calcium levels but with normal ionized calcium levels. Her evaluation was notable for the finding of an IgA monoclonal gammopathy, which paralleled her history of elevated total serum calcium. Extensive clinical investigations did not reveal hyperparathyroidism or cancer-mediated hypercalcemia. Additional in vitro analyses using relatively simple and available assays were done to compare the plasma containing the IgA paraprotein against a healthy control. These studies revealed that the plasma of the patient containing a high molecular IgA paraprotein can reduce the amount of free calcium in solution, thus providing a mechanistic explanation for the clinical observations.

## Case Presentation

**Figure 1.**
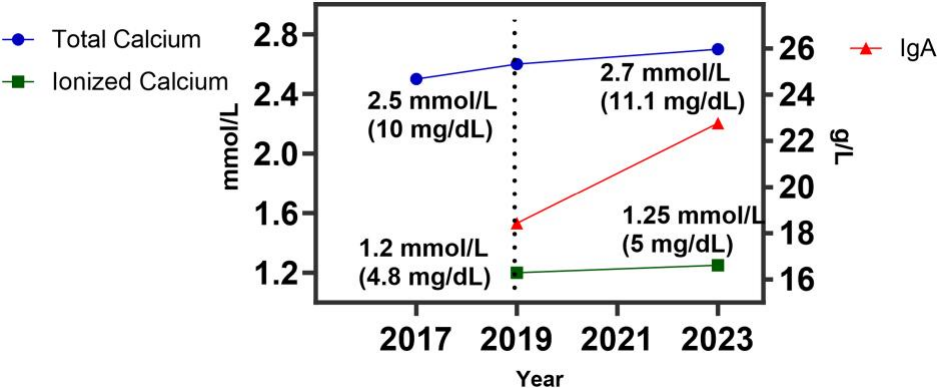
Total calcium and ionized calcium trends in the patient. The measurements of total calcium and ionized calcium over 6 years of follow are presented. The dashed line represents the time when the levels of total calcium were first noted to be abnormally high. At the same time, the IgA levels were first checked and found to be abnormal. Key values of total calcium and ionized calcium are labeled above their respective points.

A 70-year-old woman was evaluated by her primary care doctor office for hypercalcemia. During routine evaluation, the patient complained of chronic, moderate fatigue without any other localizing symptoms. She did not have fever, weight loss, gastrointestinal symptoms, numbness, or changes in her urinary habits. Her medical history was unremarkable, and she did not have chronic or recurrent infections. At the time of this evaluation, laboratory tests were notable for mild hypercalcemia, total calcium 2.6 mmol/L (10.4 mg/dL; normal reference range, 2.1-2.5 mmol/L [8.6-10.3 mg/dL]) with normal levels of serum albumin, total protein, magnesium, phosphorus bicarbonate, and estimated glomerular filtration rate based on serum creatinine. Workup for hypercalcemia showed normal levels of PTH 3.1 pmol/L (30 pg/mL; normal reference range, 1.5-6.8 pmol/L [15-65 pg/mL]), 1-25 OH vitamin D 79.2 pmol/L (33 pg/mL; normal reference range, 43.2-187 pmol/L [18-78 pg/mL]), undetectable PTH related peptide and slightly low levels of 25-OH vitamin D at 64 nmol/L (26.5 ng/mL; normal reference range, 72-192 nmol/L [30-80 ng/mL]) . Importantly, on subsequent laboratory tests, the ionized calcium was found to be normal at 1.2 mmol/L (4.8 mg/dL; normal reference range, 1.1-1.3 mmol/L [4.5-5.1 mg/dL]), whereas total calcium remained elevated at 2.72 mmol/L (10.9 mg/dL) (Fig. 1). Further work to evaluate the cause of hypercalcemia and fatigue revealed an elevated erythrocyte sedimentation rate of 71 mm/h, markedly elevated levels of immunoglobulin IgA at 21.79 g/L (2179 mg/dL; normal reference range .7-4 g/L [70-400 mg/dL]), with suppressed levels of IgG and IgM. Serum protein electrophoresis with immunofixation demonstrated an IgA Kappa paraprotein of .2 g/L (20 mg/dL).

## Diagnostic Assessment

Given the persistent elevated levels of total calcium and the presence of IgA paraprotein, additional testing was done to exclude the presence of hyperparathyroidism or cancer. Urine calcium in 24 hours was 2.1 mmol (81 mg; normal reference range, 1.2-7.5 mmol/d [50-300 mg/d]). A parathyroid gland ultrasound and scintigraphy did not reveal evidence of enlarged glands and dual energy X-ray absorptiometry scan was negative for osteoporosis (ie, there was overall no clinical evidence of hyperparathyroidism). Total body magnetic resonance imaging and photon emission tomography scan did not reveal any evidence of active lesions suggestive of malignancy or lytic lesions in the bone. A bone marrow biopsy showed hypercellular marrow with trilineage hematopoiesis and 20% to 30% involvement by plasma cell neoplasm, consistent with smoldering myeloma, not meeting criteria for multiple myeloma. Thus, the comprehensive testing and clinical course were more consistent with pseudohypercalcemia, possibly because of binding of serum calcium to IgA paraprotein [5].

**Figure 2.**
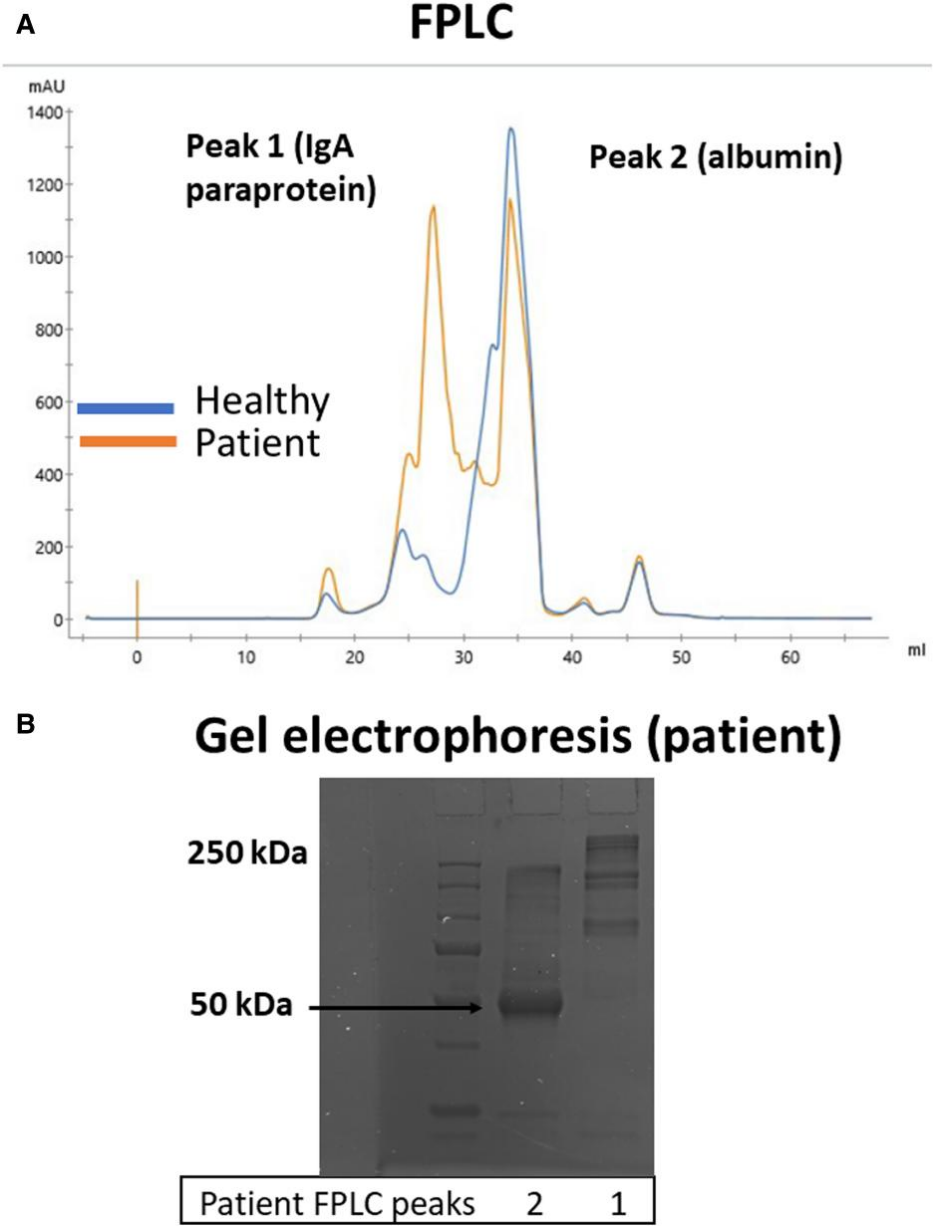
Plasma fast protein liquid chromatography (FPLC) and gel electrophoresis. (A) Plasma samples from the patient and a healthy control were diluted in PBS (800 μL of plasma in 500 μL of PBS) and separated by FPLC based on size exclusion, using ÄKTA go with a Supreose 6 Increase 10/300 GL column with a total volume of 47.1 mL at a flow rate of .5 mL/min. The Y-axis, mili-absorbance unit (mAU) reflects UV absorption of protein and varies with protein concentration. The concentration of total protein in fractions in peak 1 of the patient was ∼200 mg/dL and was ∼50 mg/dL in the same fractions of the control as measured by the BCA protein assay (Thermo scientific #23225). The elution volume, in milliliters, is presented in the X-axis. Larger size proteins migrate faster than smaller proteins and thus are represented to the left of the graph. Plasma was fractionated and collected every 500 μL. The patient FPLC demonstrated 2 clear peaks of high protein concentration, whereas the healthy control shows only 1 predominant peak, corresponding to albumin by size, against a standard. (B) Gel electrophoresis of plasma fractions in peaks 1 and 2 of the patient. Peak 2 has a MW ∼60 kDa, consistent with the expected MW of albumin, whereas peak 1 had protein bands of much higher molecular weight. Data are representative of 3 independent experiments.

To test the capacity of the patient plasma containing the paraprotein to affect the levels of Ca^++^, the patient IgA paraprotein was isolated by fractionating the plasma, based on protein size exclusion using fast protein liquid chromatography (FPLC). For reference, the plasma of a healthy adult female without hypercalcemia was tested in parallel to the patient sample. As expected from the serum protein electrophoresis results, the patient FPLC revealed 2 distinct peaks of protein corresponding in molecular weight (MW) to the presence of abnormally heavy protein (“Peak 1-IgA paraprotein”) and to albumin (“Peak 2-albumin”), respectively (Fig. 2A). The healthy control plasma fractions revealed only 1 peak, overlapping with the peak 2 of the patient, and corresponding to the MW of albumin, as revealed by gel electrophoresis (Fig. 2B) and direct comparison with a standard mix of proteins.

**Figure 3.**
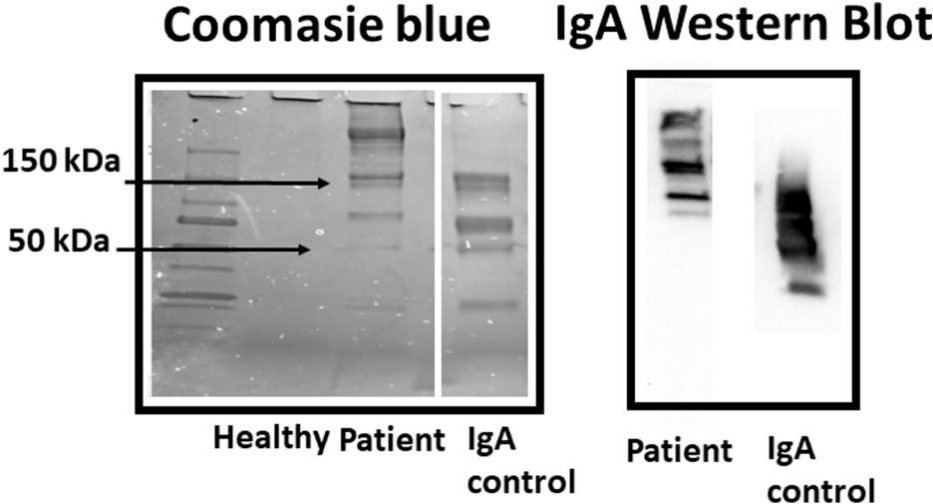
Gel electrophoresis of plasma fractions corresponding to the paraprotein peak. Corresponding plasma fractions of peak 1 derived from FPLC in the healthy control and patient were denatured with SDS and loaded at equal microliter quantities on a 4% to 20% Gel (Mini Protein Bio-Rad). After electrophoresis, the protein was visualized by Coomassie blue staining. For comparison, human plasma IgA (Sigma Aldrich Cat# 401098) was also loaded in gel. The patient plasma predominantly revealed high-molecular-weight proteins (>200 kDa) and lower amounts of protein at molecular weights of 50 kDa (ie, the approximate MW of the heavy chain of IgA). Subsequently, the same plasma fractions were denatured and loaded in gel, then transferred to a PVDF membrane, blocked for 1 hour with 5% milk and then treated with primary antibody anti-IgA heavy chain (1:1000) (Proteintech #11449-1-AP) for 3 hours at room temperature. After washing primary antibody ×3 with TBST, the membrane was treated with secondary antibody anti-rabbit-HRP (1:5000) for 30 minutes. The gels were washed ×3 with TBST and visualized using the Clarity Western ECL substrate. The results demonstrate that the majority of protein present in peak 1 of the FPLC corresponds to high-molecular-weight IgA. Data are representative of >3 independent experiments.

Gel electrophoresis and immunostaining of the abnormal peak of protein in the patient confirmed the presence of IgA in these plasma fractions (Fig. 3). Interestingly, as compared with IgA obtained commercially from the plasma of a healthy human, the patient IgA had bands at higher molecular weights (>200 kDa), likely reflecting the presence of polymers of IgA, which resisted denaturization with sodium dodecyl sulfate (Fig. 3) [5].

**Figure 4.**
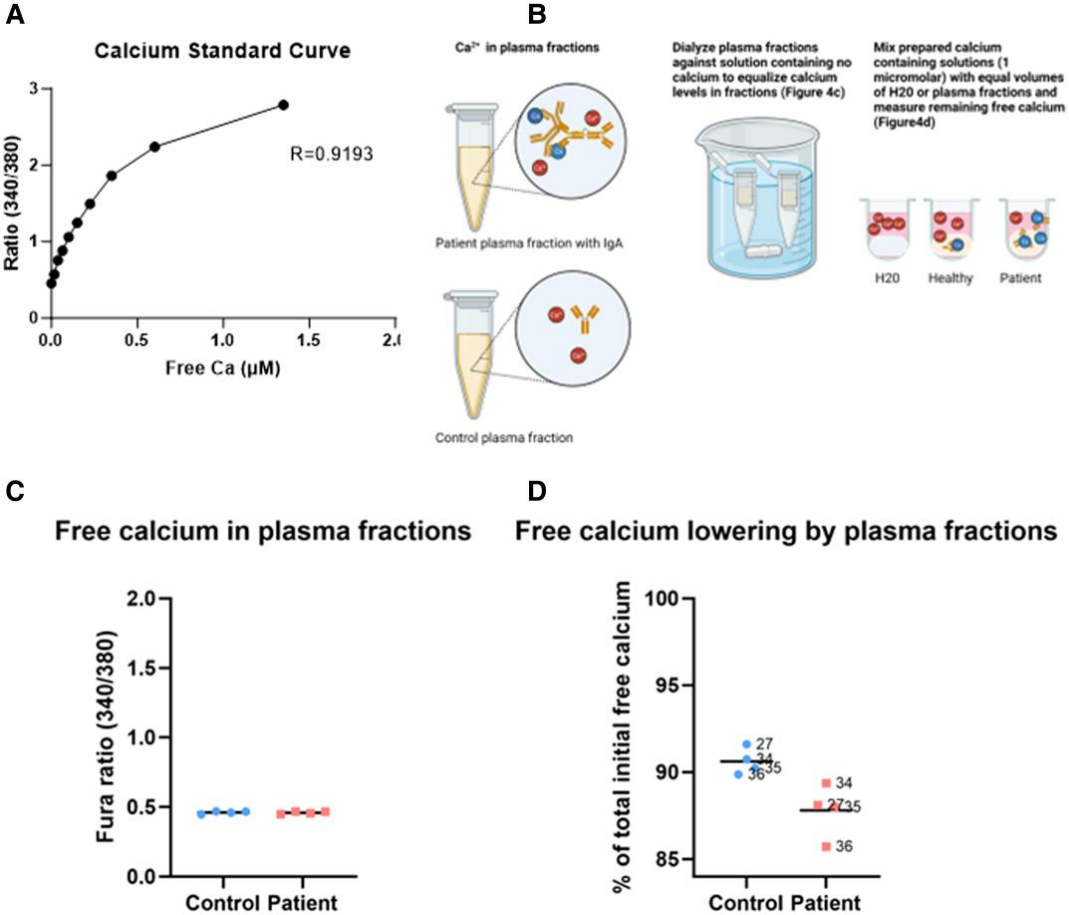
Free calcium measurement in plasma fractions. (A) A calcium standard curve was created using a calcium calibration kit (Biotium #59100) to contain levels of free calcium in the 1 to 1 μM range at a pH of 7.4. Levels of free calcium were measured by mixing 1 μL of Fura Pentasodium (1 mM) with 200 μL of calcium solution. Fluorescence intensity in the solutions was measured by sequential excitations at 340 and 380 nm, with emission at 510 nm using a 96-well black plate and a BioTek Synergy H1 Plate reader. An increase in the 340/380 ratio is noted with free calcium concentrations going up to 1 μM. (B) Workflow of experiments to measure free calcium in plasma fractions. Free calcium (Ca^++^) is represented as calcium not in direct contact with immunoglobulins. The patient plasma fraction had high amounts of IgA (see Figs. 2 and 3), whereas the control had a lower amount of protein (see Fig. 2) in the corresponding fractions., Both plasma fractions were dialyzed against PBS (no calcium) to remove the endogenous Ca^++^ and equalize levels of free calcium at baseline. Plasma fractions were then added to solutions with a known concentration of free calcium (1 μM) and the remaining free calcium was measured. The figure was created using BioRender. (C) Plasma fractions were dialyzed using a membrane with cutoff molecular weight 3.5 kDa (Sigma Aldrich Cat#PURX35005) in a PBS (no calcium, no magnesium) solution overnight. Free calcium was measured in the fractions by Fura Pentasodium after dialysis to provide a baseline at which the levels of free calcium were similar in the patient-derived fractions of peak 1 (IgA paraprotein) vs healthy control. Baseline measurements were done using 100 μL of post-dialysis fraction + 200 μL of “zero calcium solution” from the calcium calibration kit. (D) A 200-μL solution containing 1 μM of free calcium was mixed with 100 μL of the plasma fractions or with equal volume of H_2_0. Mixtures of calcium solution and plasmas (or H_2_0) were centrifugated (20 000 RCF ×5 minutes) and the supernatant tested for free calcium by addition of 1 μL Fura pentasodium and measured as in panel A. Each patient plasma fraction containing IgA lowered the amount of free calcium to a greater than each corresponding fraction in the healthy control. The results demonstrate a modest (∼4% decrease), yet consistent capacity of the patient IgA containing fractions to lower free calcium in solution vs healthy control plasma. For 1:1 comparison between patient and heathy plasma fractions of equivalent molecular weight, the number of the fraction tested is provided.

To evaluate the possible calcium-lowering properties of the patient plasma fraction containing IgA, we used the ratio metric calcium indicator Fura-2 Pentasodium (Biotium, catalog No. 50032), which detects changes in free calcium between the range of 1 nM to 1 μM at physiologic pH (Fig. 4A) [6]. In contrast to Fura-2 2 acetoxymethyl ester, which is typically used to measure intracellular free calcium, Fura-2 pentasodium is cell impermeant and has been shown to have good correlation with potentiometric techniques to measure free calcium in different solutions [6]. Of note, total levels of calcium using a colorimetric detector (Stanbio Laboratory, catalog No. 0150) were too low for detection in each of the plasma fractions and are thus not reported.

The workflow of in vitro experiments is illustrated in Fig. 4B. To reduce endogenous calcium present in the fractions and generate similar baseline level of free calcium, the plasma fractions were dialyzed against phosphate-based saline (without calcium) using dialysis membranes with a molecular weight cutoff of 3.5 kDa (Fig. 4C). To then assess the capacity of protein in the plasma fractions to lower calcium, we mixed the plasma fractions 1:2 with a solution containing 1 μM of calcium, and the amount of free calcium that remained after mixing with the plasma fractions was determined. Relative to the amount of free calcium without addition of the plasma fractions (100%), each of the patient plasma fractions containing the IgA paraprotein reduced free calcium more than the fraction with corresponding MW in the healthy control (Fig. 4D) The results thus support increased capacity of the patient IgA paraprotein to lower calcium relative to proteins of similar molecular weight in a control, which were much less abundant.

## Treatment

Together, the clinical findings of persistent disproportional increase in total calcium vs ionized calcium, elevated IgA, lack of secondary causes of hypercalcemia, along with the evidence of high-MW IgA paraprotein with free calcium-lowering capacity, supported the conclusion that the physiologically relevant free calcium was not altered but rather that there was increased capacity of the patient plasma proteins to bind calcium. Thus, no specific treatment for hypercalcemia was given at this point.

## Outcome and Follow-up

The patient continues to follow regularly with internal medicine and oncology. She remains asymptomatic and without evidence of end-organ damage from the paraprotein or hypercalcemia.

## Discussion

Normal levels of free calcium are critical for cellular function. More commonly, disorders of calcium homeostasis are encountered in patients with parathyroid hormone diseases or their regulators and effectors such as the calcium-sensing receptor and vitamin D [1]. Total levels of calcium are routinely measured in chemical panels and often used to diagnose hypo- or hypercalcemia. Clinicians are usually aware that low levels of total calcium need to be interpreted in the context of serum albumin levels. However, high levels of total calcium concomitant with normal levels of ionized calcium are less commonly encountered or suspected.

Serum albumin is the most abundant protein and is negatively charged, and thus is able to bind total calcium. Other negatively charged proteins such as immunoglobulins may also bind calcium but proportionally much less than albumin, except in certain cases where they are clonal or abnormally elevated [5, 7]. In addition to paraproteins, other causes of pseudohypercalcemia include hyperalbuminemia, thrombocytosis, and myeloproliferative disorders [8]. In this case, the patient's IgA levels were elevated to the same extent as her albumin levels and correlated with the discrepant findings between the total calcium and ionized calcium levels. IgA is the predominant immunoglobulin found in mucosal sites, where its typically found in a dimeric or polymeric forms [9]. In contrast, IgA is normally less abundant than IgG in serum and is predominantly monomeric in circulation [9]. In our case, we found evidence of high levels of a circulating IgA, which appeared to be predominantly circulating in a polymeric form. In addition, the high levels of the protein and/or specific physicochemical properties likely allow it to lower free calcium in solution.

Previous cases had documented the capacity of paraproteins to bind calcium. Some of these studies used radiolabeled calcium to provide important insights about the relative capacity of these immunoglobulins to bind calcium [5, 10]. However, there is limited availability of assays involving radiolabeled compounds, especially in the clinical setting, and thus additional more readily accessible forms to evaluate free calcium and its changes in relation to patient protein are important. We chose to use Fura Pentasodium free calcium indicator given that is a well-characterized, available assay with good sensitivity to detect changes at low molar concentrations of calcium [6]. Furthermore, we compared the plasma of the patient and a healthy control at similar volumetric quantities, rather than attempting to normalize the very high levels of protein in the patient to that of the healthy control. This is because the clinical question posed was the capacity of each plasma to lower calcium, with its intrinsic protein concentrations. However, our methodology does not allow us to exclude an assay interference by the paraprotein causing pH changes or turbidity, artificially elevating total calcium results. We also do not provide a direct measurement of any of the physicochemical properties of this IgA paraprotein and/or the mechanisms by which it may bind calcium.

Our case highlights the importance of checking ionized calcium levels in any patient with suspected or diagnosed alteration in calcium metabolism, including hypercalcemia in patients with premalignant or malignant conditions, and to interpret calcium levels in the context of the clinical setting and changes in other proteins beyond albumin.

## Learning Points

- Ionized calcium levels should be measured in all patients with abnormal levels of total calcium.
- Immunoglobulins can affect the proportional levels of total and ionized calcium.
- When evaluating a patient with smoldering myeloma or multiple myeloma, the possibility of pseudohypercalcemia should be considered.

## Data Availability

Original data generated and analyzed during this study are included in this published article.

## Abbreviations

Ca^++^: ionized calcium
FPLC: fast protein liquid chromatography
MW: molecular weight
